# Infusion of blood from mice displaying cerebral amyloidosis accelerates amyloid pathology in animal models of Alzheimer’s disease

**DOI:** 10.1186/s40478-020-01087-1

**Published:** 2020-12-07

**Authors:** Rodrigo Morales, Claudia Duran-Aniotz, Javiera Bravo-Alegria, Lisbell D. Estrada, Mohammad Shahnawaz, Ping-Ping Hu, Carlos Kramm, Diego Morales-Scheihing, Akihiko Urayama, Claudio Soto

**Affiliations:** 1 Mitchell Center for Alzheimer’s Disease and Related Brain Disorders, Department of Neurology, The University of Texas Medical School at Houston, Houston, TX 77030 USA; 2 Centro Integrativo de Biología y Química Aplicada (CIBQA), Universidad Bernardo O’Higgins, Santiago, Chile; 3 Center for Social and Cognitive Neuroscience (CSCN), School of Psychology, Universidad Adolfo Ibáñez, Diagonal Las Torres, 2640, Santiago, Chile; 4 Facultad de Medicina, Universidad de los Andes, Av. San Carlos de Apoquindo 2200, Las Condes, Santiago Chile; 5 Facultad de Ciencias de la Salud, Universidad Bernardo O’Higgins, Santiago, Chile; 6 Chongqing Key Laboratory of Natural Product Synthesis and Drug Research, School of Pharmaceutical Sciences, Chongqing University, Chongqing, 401331 China

**Keywords:** Amyloid-beta, Blood transfusion, Prions

## Abstract

Previous studies showed that injection of tissue extracts containing amyloid-β (Aβ) aggregates accelerate amyloid deposition in the brain of mouse models of Alzheimer’s disease (AD) through prion-like mechanisms. In this study, we evaluated whether brain amyloidosis could be accelerated by blood infusions, procedures that have been shown to transmit prion diseases in animals and humans. Young transgenic mice infused with whole blood or plasma from old animals with extensive Aβ deposition in their brains developed significantly higher levels brain amyloidosis and neuroinflammation compared to untreated animals or mice infused with wild type blood. Similarly, intra-venous injection of purified Aβ aggregates accelerated amyloid pathology, supporting the concept that Aβ seeds present in blood can reach the brain to promote neuropathological alterations in the brain of treated animals. However, an amyloid-enhancing effect of other factors present in the blood of donors cannot be discarded. Our results may help to understand the role of peripheral (amyloid-dependent or -independent) factors implicated in the development of AD and uncover new strategies for disease intervention.

**Electronic supplementary material:**

The online version of this article (10.1186/s40478-020-01087-1) contains supplementary material, which is available to authorized users.

## Introduction

Alzheimer’s disease (AD) is a fatal neurodegenerative disorder which represents the most common cause of dementia in the elderly population. Currently, there is no effective treatment for this disorder. Despite decades of research, the etiology of the large majority of AD cases is still largely unknown. A hallmark neuropathological feature of AD includes the presence of cerebral deposits of fibrillar aggregates consisting of amyloid plaques and neurofibrillary tangles [1]. Compelling evidence suggest that misfolding and aggregation of Aβ might be the triggering event, responsible for inducing brain abnormalities in this disease [2]. Although the molecular bases of AD have been extensively studied, the first events activating pathological changes still remain unknown in most of the cases. AD is a heterogeneous and multifactorial disease and its underlying etiology is largely unclear. A small proportion of AD cases, so called familial AD, are linked to mutations in genes encoding the amyloid precursor protein (APP) and presenilins (PS1 and PS2). Mutations in these genes result in elevated levels of Aβ, which become deposited in the brains of AD patients as senile plaques. Nevertheless, sporadic cases of AD represent around 95% of all cases. Specific events triggering sporadic disease are unknown, but there are several risk factors that can increase the probabilities to develop AD [3].

Interestingly, the accumulation of misfolded protein aggregates is the predominant pathological feature of several other diseases known collectively as Protein Misfolding Disorders (PMDs). PMDs includes several brain and systemic maladies such as AD, Parkinson’s disease, amyotrophic lateral sclerosis, Huntington’s disease, type-2 diabetes, systemic amyloidosis and Transmissible Spongiform Encephalopathies (TSEs) or prion diseases [4]. Despite the fact that each PMD is characterized by the deposition of aggregates formed by different proteins (Aβ and tau in AD, prion protein in TSEs, α-synuclein in Parkinson’s disease, amylin in diabetes type 2, etc.), they share many morphological, biological and biochemical features [4]. PMDs can have sporadic or inherited origins, except in the case of prion diseases, in which the pathology can be also transmitted by infection [5]. Strikingly, the infectious agent responsible for prion diseases (termed prion) is mostly composed by the misfolded and aggregated form of the prion protein that has the ability to propagate the disease through an infectious process [5]. Prion replication depend on the auto-catalytic conversion of the normal prion protein (termed PrP^C^) catalyzed by small amounts of the misfolded and infectious form of the prion protein (termed PrP^Sc^). The conversion of PrP^C^ into PrP^Sc^ follows a seeding-nucleation mechanism, in which oligomers of the misfolded protein bind, induce the misfolding and integrate the normal protein into the growing aggregates [6, 7]. The seeding-nucleation model of prion propagation provides a plausible mechanism to explain prion infectivity and has been reproduced in vitro to “cultivate” prions with infectious properties when inoculated into animals [8].

Since the mechanism of prion replication is remarkably similar to the process of amyloid formation, we and others have hypothesized that misfolded aggregates associated to AD and other PMDs can spread by the prion principle [7]. Remarkably, a series of reports, using cellular and/or animal models, have provided evidence suggesting that the transmission of protein misfolding by a prion-like mechanism might be at the heart of the most common PMDs, including Alzheimer’s and Parkinson’s diseases [9, 10]. In the specific case of AD, various studies in animal models have shown that injection of AD brain extracts can induce or accelerate Aβ deposition in the brain through a prion principle of spreading and transmission of misfolded aggregates [11–14]. However, an important difference between these prion-like transmissible events and the *bona*-*fide* prion phenomenon associated to TSEs is the lack of evidence for natural transmission for any of the other PMDs. Furthermore, TSE prions can produce disease when administered by various routes, including oral, intra-venous, intra-peritoneal, nasal, intra-ocular and even through aerosols [5]. Finally, infectious prions have been detected in many peripheral organs, circulating in blood, lymphatic fluid and cerebrospinal fluid, as well as secreted in urine, saliva and feces [5]. The widespread tissue distribution of infectious prions and the diversity of routes by which they can produce infection make natural transmission much more feasible. So far, most of the experiments to assess spreading and transmission of non-prion misfolded proteins have been done by direct intra-cerebral administration of brain homogenates containing large concentrations of misfolded proteins. One interesting study in this regard was the demonstration that cerebral accumulation of Aβ deposits can be accelerated by intra-peritoneal inoculation of transgenic mice with amyloid-rich brain extracts [13]. This finding suggests that, analogous to prions, Aβ seeds administered by peripheral exposures may induce disease in the brain. However, the source of misfolded Aβ used in these experiments was sick brain homogenates. Recently, additional reports have further supported the important role of circulating Aβ on brain pathology. Bu and colleagues showed that wild-type mice can develop brain amyloidosis after parabiosis to transgenic mice overexpressing mutated versions of APP and presenilin-1 [15]. Another report suggests that intra-venous (i.v.) injection of brain homogenates containing Aβ aggregates promote the appearance of cerebral amyloid angiopathy [16]. To evaluate whether the induction of amyloid deposition can be also observed under more natural conditions, we studied the pathological transmission through blood transfusion, a medically relevant route that has been shown to transmit prion disease in animals and humans [17, 18].

## Materials and methods

### Animals

Experiments described in this article utilized two different transgenic models of amyloidosis in AD: Tg2576 [19] and APP/PS1 mice [20]. Tg2576 mice express the human amyloid precursor protein (APP) harboring the Swedish mutation. As a result, these mice start to develop Aβ deposits in their brains at 8–9 months old and extensive presence of senile plaques and inflammation at 17 months of age. APP/PS1 mice express the human APP with the Swedish mutation and the human presenilin-1 protein with the DeltaE9 mutation which is associated with a form of early-onset AD. As a result, APP/PS1 mice show occasional presence of Aβ aggregates as early as 4–5 months old and extensive amyloid plaque accumulation by 12 months of age. 4–13 animals (random mixtures of males and females) were used per experimental group as indicated in the figure legends. Sex distribution of mice in each group is shown in Additional file 1: Table S1.

### Preparation of recombinant Aβ aggregates

Lyophilized recombinant Aβ was resuspended in 0.1% NH_4_OH. At the time of use, pH was neutralized with 2X PBS reaching 1 mg/mL final protein concentration. This sample was incubated for 5 days at 25 °C with shaking at 500 rpm in an Eppendorf^®^ thermomixer. Finally, this preparation was diluted by adding one volume of 1X PBS. Formation of aggregates was confirmed by Thioflavin T binding assay and electron microscopy, as described [21].

### Blood and brain collection from old transgenic and wild type mice

Transgenic mice used as blood donors were sacrificed by CO_2_ inhalation at the ages indicated in Table 1. Approximately 1 mL of blood was collected per animal by cardiac puncture using 26G 3/8 needles (BD Biosciences, Franklin Lakes, NJ) and syringes containing 50 µL of 1 mg/mL heparin solution (Sigma-Aldrich, St. Louis, MO). Samples were immediately injected into mice as described below. Blood from WT mice was obtained in the same way from non-transgenic littermates of the previously mentioned donors. For the experiments using separated blood fractions, whole blood was pooled according to the transgene and centrifuged at 300 × g for 10 min. Each preparation resulted in approximately 40% of plasma and 60% of blood cells suspension. Plasma and cells were separated and volume was completed to a 100% using sterile PBS. Brain homogenate from a 12 months old Tg2576 mouse was prepared at 10% w/v in PBS.

**Table 1:** Summary of experiments

	Inoculum	Recipient	Attack rate	Percentage of animals affected
Experiment 1^a^	12–14 months old Tg2576 blood	Tg2576	5/5	80–100%
Experiment 2^b^	12–14 months old Tg2576 blood cells or plasma	Tg2576	0/4 (cells) 4/6 (plasma)	0% (cells) 66.7% (plasma)
Experiment 3^c^	Recombinant Aβ fibrils	APP/PS1	5/6	83.3%
Experiment 4^d^	12 months old APP/PS1 blood	APP/PS1	4/5	80%
Experiment 5^d^	2–4 months old Tg2576 blood	Tg2576	1/8	12.5%
Experiment 6^d^	7–9 months old Tg2576 blood	Tg2576	3/7	42.9%
Experiment 7^d^	15–20 months old Tg2576 blood	Tg2576	1/6	16.7%
Experiment 8^e^	10% brain homogenate from 12 months old Tg2576	Tg2576	2/2	100%

^a^Detailed data for this experiment is shown in Figs. 1, 2 and 3. In the table, results of only two transfusions are included

^b^Detailed data for this experiment is shown in Fig. 5

^c^Detailed data for this experiment is shown in Fig. 6

^d^Detailed data in Additional figures

^e^Only 2 of the 20 injected mice survived to the experimental endpoint

### Intra-venous blood treatment and intra-cerebral injections

For experiments involving Tg2576 mice, 50 days-old transgenic mice or WT littermates were anesthetized with 2,2,2-tribromoethanol and injected with 150 µL of 12–14 months old Tg2576 whole blood, 300 µL of blood components or the same materials from WT animals in the tail vein using a ½ cc 27G ½ tuberculin syringe (BD Biosciences, Franklin Lakes, NJ). Animals receiving two doses of either Tg or WT blood or blood components were submitted to the same procedure at 80 days old. Tg2576 mice receiving blood from 2–4, 7–9 to 15–20 months old donors were injected in the jugular vein using the same schedule described above with an additional injection at 57 days old and sacrificed at 300 days old. For blood transfusions in APP/PS1 mice, 60 days old transgenic mice were anesthetized and 200 µL of blood from either old transgenic animals or age-matched WT littermates were injected through the tail vein, once a week, 3 times in total. For intra-cerebral injections of brain homogenates, 55 days old Tg2576 mice were injected with 5 µL of either 10% brain homogenate or PBS in the right hippocampus using the following stereotaxic coordinates: anterioposterior (AP) = − 1.8 mm; mediolateral (ML) = − 1.8 mm; dorsoventral (DV) = − 1.8 mm. Mice receiving i.v. infusions of brain homogenates were anesthetized as described above and injected at 50 days old with 150 µL of a 10% w/v extract prepared in PBS (brain source: 12 months old Tg2576 mouse). For i.v. injections of recombinant Aβ aggregates, 30 days old APP/PS1 mice were anesthetized as described above and injected in the jugular vein with 200 µL of a preparation containing 0.5 mg/mL of the peptide in a fibrillar form. This procedure was repeated weekly for two consecutive weeks. Animals were monitored until recovery and carefully observed during the following days in order to check for any possible complications related to the procedures. Experimental endpoints for this study was ~ 250 or 300 days old for Tg2576 mice (as described above), and 150 (blood transfusions) or 180 (recombinant Aβ infusions) days old for APP/PS1 mice. A summary of all experiments involving i.v. treatments is provided in Additional file 1: Figure S1.

### Evaluation of systemic inflammatory response after blood infusion

50-days-old Tg2576 mice and WT littermates were i.v. injected with 150 µL of WT or Tg2576 blood from 12 to 14 months old mice. Groups of animals (n = 4) were sacrificed 1.5 h or 7 days after the treatment whereas others were sacrificed 1.5 h or 7 days after a second blood transfusion occurring 30 days later. Plasma and peripheral blood mononuclear cells (PBMC) were obtained from blood of treated and non-treated Tg2576 and WT mice at the indicated times post-transfusion. Plasma and PBMC were isolated by Ficoll-paque gradient separation (GE Lifesciences, Little Chalfont, UK). 1 × 10^6^ PBMC/mL were incubated for 15 h with 100 ng/mL of lipopolysaccharides from *Escherichia coli* (LPS, Sigma-Aldrich, St. Louis, MO) diluted in RPMI Medium 1640 (Sigma-Aldrich, St. Louis, MO) and supplemented with 10% fetal bovine serum. Supernatants were recovered and stored at − 80 °C until use. IL-1β, IL-6, TNF-α, MIP-1α, IFN-γ, IL-10 and MCP-1 in plasma and PBMC supernatants were measured by the respective Quantikine Kit (R&D Systems, Minneapolis, MN), according to manufacturer’s specifications. Signals were read on an EL800 BIO-TEK ELISA plate reader (BioTek, Winooski, VT) at 450 nm. The same experimental procedures were performed in animals injected with different blood components (n = 4–6). Samples in this case were obtained at the experiment’s endpoint (~ 200 days after the first inoculation).

### Histological analyses of brain slices

Tissue staining was performed as previously described [11] with minor modifications. Briefly, brain was collected. Half-brain (left) was frozen at − 80 °C for biochemical analyses whereas the other half (right) was stored in formalin for histological studies. Formalin stored tissues were paraffin embedded and cut at 8–10 μm. Slices were then incubated overnight at 4 °C with primary antibodies: 4G8 monoclonal antibody (1:1000) (Covance, Princeton, NJ), anti-GFAP antibody (1:1000) (Abcam, Cambridge, MA) or anti-Iba1 antibody (1:1000) (Wako Chemicals, Japan). Tissue slices were washed with PBS and incubated with the following secondary antibodies: sheep anti-mouse IgG-HRP or goat anti-rabbit IgG-HRP (1:1000) (RPN4201 and RPN4301, GE Healthcare, Little Chalfont, UK). Samples were developed with DAB (Sigma-Aldrich, St. Louis, MO) at room temperature. For ThS staining, slides were placed in 0.025% aqueous ThS (Sigma-Aldrich, St. Louis MO) in 50% alcohol solution. After that, slices were stained with Mayer’s Hematoxylin. Finally, the samples were mounted with Super Mount (Innogenex, San Ramon, CA) and cover slips. Brain slices from APP/PS1 injected with recombinant Aβ were treated with 85% formic acid for 5 min after tissue hydration. All other specimens were not submitted to this treatment. Samples were analyzed using a Leica DMI6000 B microscope and subjected to image analysis using the ImagePro software. Lateromedial sagital slides of mice brains from different experimental groups were quantified for ThS staining and anti-Aβ, -GFAP and -Iba-1 immunostaining. Aβ and ThS were quantified in every fifth section (with a distance of 400 μm) in a total of 11 sections per animal. The analysis of many slides is important to detect the effect, which as explained in the text, is much lower than that generally observed when brain homogenate is injected directly into the brain. GFAP and Iba-1 burdens were quantified in every tenth section (with a distance of 400 μm) in a total of 6 sections per animal. Burden was defined as the labeled area of the brain per total area analyzed and is expressed as a percentage. The region analyzed corresponded to the entire cortical and hippocampal areas of the sections studied. Both the histological staining and image analyses were performed in a blinded fashion.

### Aβ_40_ and Aβ_42_ quantification by ELISA

Brain Aβ_40_ and Aβ_42_ levels were measured in 10% brain homogenates prepared in PBS plus Protease Inhibitors Cocktail (Roche Diagnostics GmbH, Mannheim, Germany). 600 µL (200 µL per tube) of PBS prepared brain homogenate were centrifuged in a L100K ultracentrifuge (Beckman-Coulter, Brea, CA) at 32,600 rpm for 1 h at 4 °C in a 42.2 Ti rotor. Supernatants were collected and pellets were resuspended in 200 µL of 70% Formic Acid (Fisher Scientific, Waltham, MA). Samples were centrifuged for 30 min at the same conditions described above and supernatants were collected. Formic acid fractions were diluted 20 times in 1 M Tris buffer, pH 11 (Sigma-Aldrich, St. Louis, MO) in order to neutralize the pH. Aβ_40_ and Aβ_42_ levels in PBS and formic acid extracts were measured by using Human Aβ ELISA Kits (KHB3482 and KHB3442 respectively, Invitrogen, Carlsbad, CA), according to manufacturer’s recommendations. Samples were read on an ELISA reader (EL800 BIO-TEK, BioTek, Winooski, VT) at 450 nm. The person performing these studies was blinded to the treatment applied to the samples. Experiments with APP/PS1 mice were performed in the same way but using 200 µL of brain homogenate as starting material.

### Statistical analyses

Data were expressed as mean ± standard error of the mean (SEM). After confirming normal distribution with Skewness/Kurtosis statistic test, one way ANOVA followed by a Tukey’s multiple comparison test was used to analyze differences between experimental and control groups. Statistical analyses were performed using Graph Pad Prism 5.0 software. Statistical differences were considered significant for values of *P* < 0.05. Attack rates were defined as the ratio of mice exhibiting an extent of amyloid pathology that was higher than 2 standard deviations over the average of the control group.

## Results

### Acceleration of amyloid pathology by blood infusion

**Fig. 1:**
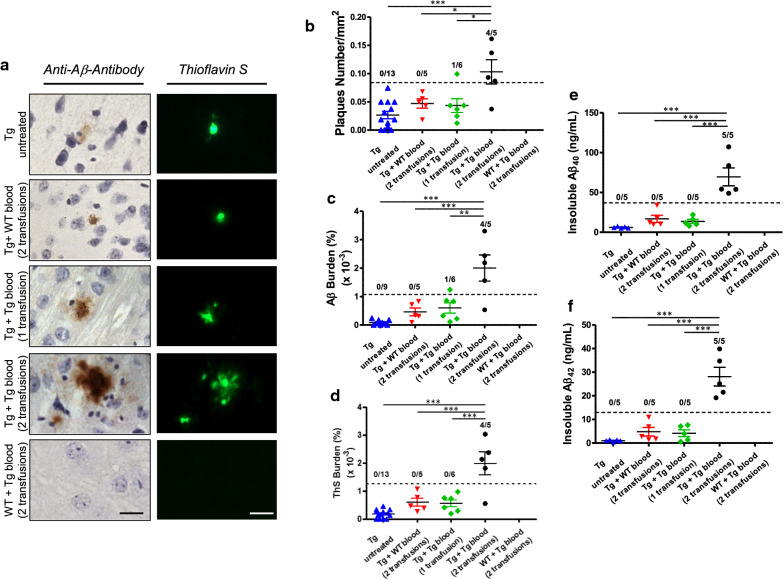
Infusion of blood from old Tg2576 mice accelerates Aβ deposition in young mice. **a** Representative pictures of amyloid deposits in the cortical area stained with the 4G8 anti-Aβ antibody and Thioflavin S (ThS). The scale bar corresponds to 20 µm. **b** The number of Aβ reactive plaques was counted and expressed as number of plaques per mm^2^ of brain area analyzed. **c** The area of antibody-reactive Aβ deposits in each group was measured and divided by the total brain area (hippocampus and cortex region) analyzed (Aβ burden). The values are expressed as a percentage and multiplied by 1000. **d** The signal of ThS reactive deposits was measured as the stained area in relation to the total brain area (hippocampus and cortex region) analyzed (ThS burden) and was expressed as percentage. The quantity of insoluble Aβ_40_ (**e**) and Aβ_42_ (**f**) was measured using ELISA kits designed to specifically detect each of these variants. The values showed in the graphs displayed in panels **b**–**f** are expressed as mean ± SEM of the different animals used in each group (n = 5–6). For statistical analysis we did not consider the group of wild type animals injected with Tg2576 blood, since all these values were 0. *P < 0.05; **P < 0.01; ***P < 0.001 based on ANOVA followed by the Tukey’s multiple comparison post hoc test. In panels **b**–**f** the dotted line represents the threshold value used to calculate attack rate, i.e. the proportion of individual animals having significantly higher pathology than controls as explained in “Materials and methods” section. The numbers on top indicate the ratio of animals over the threshold/total number of animals in the group

For most of our experiments we used Tg2576, a transgenic mouse line expressing the human APP gene harboring the Swedish mutation [19]. These animals produce soluble Aβ with the human sequence, and develop typical senile plaques which are detectable starting at ~ 8–9 months of age. Groups of 50 days old Tg2576 mice (n = 5–6) were intravenously (i.v.) infused with 150 µL of whole blood from 12 to 14 months old Tg2576 mice (Tg-blood) in order to assess a putative acceleration of brain amyloidosis by blood infusion. Mice were treated with either one or two whole-blood transfusions (separated by 30 days) with the purpose of analyzing dose-dependency. As controls, a group of young Tg2576 mice was subjected to 2 transfusions with blood from age-matched wild type non-transgenic littermates (WT-blood) and a group of young wild type non-transgenic littermates was infused twice with the same Tg-blood used for the experimental groups. All animals were sacrificed at approximately 250 (between 248 and 254) days of age and brains were collected for histopathological and biochemical analyses. Amyloid deposits were observed by histological staining with the 4G8 anti-Aβ antibody and Thioflavin S (ThS) mainly in the hippocampal and cortical areas (Fig. 1a). The extent of amyloid deposition was compared among all different control and experimental groups and measured in terms of the number of deposits per unit of area (Fig. 1b), the area of the brain occupied by Aβ deposits reactive against an anti-Aβ antibody (Fig. 1c) and the area of the brain occupied by ThS reactive fibrillar amyloid plaques (Fig. 1d). Strikingly, the amyloid load measured by any of these three ways was significantly higher in Tg2576 animals subjected to 2 transfusions with blood coming from old transgenic mice as compared to untreated Tg2576 or controls infused with WT-blood. In contrast, no statistically significant increase of amyloid deposition was observed in Tg2576 mice subjected to only 1 blood transfusion, indicating that the effect requires either a higher quantity of material and/or a repeated administration. The histological results were further corroborated by biochemical measurements of the pool of aggregated Aβ following formic acid extraction and Aβ quantification by ELISA. The levels of both insoluble Aβ_40_ (Fig. 1e) and Aβ_42_ (Fig. 1f) were > fourfold higher in Tg2576 treated with 2 blood transfusions as compared with all the other groups. In addition to measure the significance of the effects observed in the different groups, we also expressed the results in Fig. 1 as attack rates, which represent the proportion of the total animals per group in which the pathology was significantly higher than in controls. This analysis was inspired by the well-established finding that only a fraction of experimental animals receiving i.v. administration of prion contaminated blood develop pathology [22, 23]. In our experiments, we defined attack rate as the ratio of mice exhibiting an extent of amyloid pathology that was higher than 2 standard deviations over the average of the control group treated with wild type blood. As shown in Fig. 1, the attack rate for the group treated with 2 Tg-blood transfusions was between 80 and 100%, depending on the parameter measured.

**Fig. 2:**
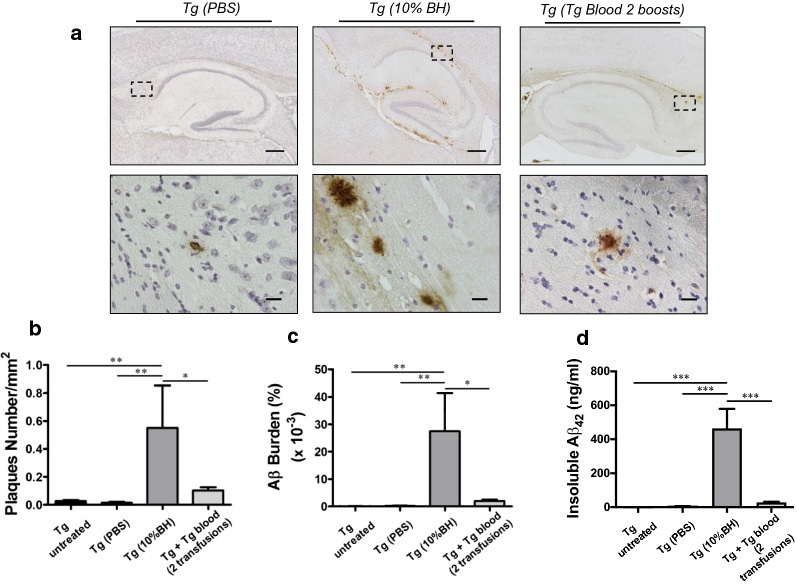
Intra-cerebral injection of brain homogenate from an old Tg2576 mouse into young mice induces substantial Aβ deposition. **a** Representative pictures of 4G8 stained brain slices from Tg2576 (n = 4–5) intra-cerebrally injected with PBS or 10% brain homogenate obtained from a 12 months old Tg2576 mouse harboring amyloid plaques (left and middle panels, respectively). Two different magnifications are shown. The scale bars in top and bottom panels correspond to 300 μm and 20 μm, respectively. The dashed squares in top panels represent the area depicted in bottom panels. The picture in the right corresponds to an animal subjected to 2 transfusions with Tg-blood (Experiment 1). The magnified image in “Tg (10% BH)” was obtained from a consecutive brain slice and display the same highlighted plaques. **b** Number of Aβ reactive plaques, which was expressed as number of plaques per mm^2^ of the brain area analyzed. **c** The area of antibody-reactive Aβ deposits in each group was measured and divided by the total brain area analyzed (Aβ burden). The values are expressed as a percentage and multiplied by 1000. **d** The quantity of insoluble Aβ_42_ was measured using ELISA kits after formic acid extraction, as described in “Materials and methods” section. For panels **b**–**d**, values on untreated animals are shown as reference. The values showed in the graphs are expressed as mean ± SEM of the different animals used in each group. *P < 0.05, **P < 0.01, ***P < 0.001 based on ANOVA followed by the Tukey’s multiple comparison post hoc test

Although the extent of amyloid deposition in the group of animals subjected to 2 Tg-blood transfusions was substantially higher compared to other groups, it was much lower than the Aβ deposition induced by direct intra-cerebral (i.c.) injection of a brain extract (Fig. 2). Indeed, the number and burden of amyloid deposits as well as the quantity of insoluble Aβ_42_ was many folds higher in animals subjected to direct i.c. administration of brain homogenate compared with those receiving 2 blood transfusions (Fig. 2). This is not surprising, considering that AD is a brain disease and that even higher differences on transmissibility between i.c. and i.v. routes have been observed for infectious prions [24, 25].

### Blood transfusion induces associated neuropathological alterations

**Fig. 3:**
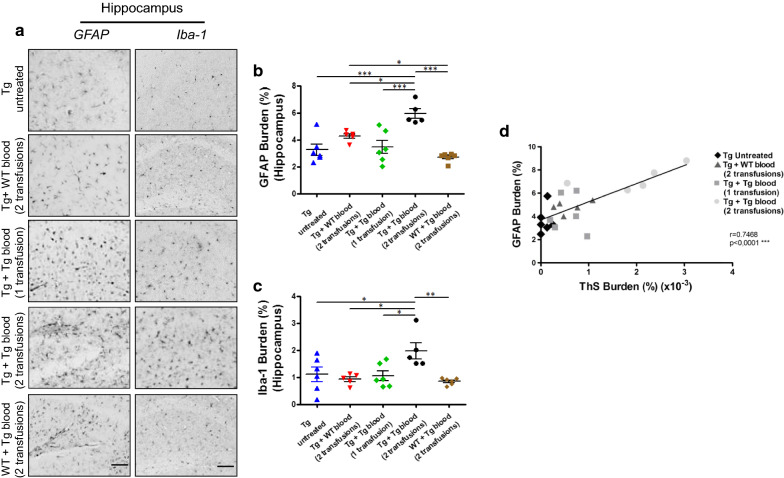
Brain inflammation in Tg2576 mice is increased by infusion of Tg-blood. **a** Representative pictures of reactive astrocytosis and microglial activation observed in the hippocampus of different animals. The scale bar represents 300 µm. Image analysis was done to quantify the extent of astrocytosis (**b**) and microgliosis (**c**) in different groups. The values correspond to the area stained with GFAP or Iba-1 antibodies per unit of brain area analyzed and are expressed as a percentage (mean ± SEM) (n = 5–6). *P < 0.05; **P < 0.01; ***P < 0.001 based on ANOVA followed by the Tukey’s multiple comparison post hoc test. **d** Correlation between the extent of amyloid deposition measured by the ThS burden (as shown in Fig. 1d) and GFAP burden in each of the individual animals studied in the groups of Tg2576 untreated or subjected to 1 or 2 transfusions with blood from old Tg2576 or to 2 transfusions with wild type blood

We also studied whether the increase of Aβ accumulation induced by blood infusions was associated with other neuropathological features. A typical alteration in AD, associated with the accumulation of amyloid plaques, is brain inflammation, which appears in the form of reactive astrocytes and activated microglia. For that reason, we stained hippocampal sections from experimental and control animals with antibodies against the glial fibrillary acidic protein (GFAP) and the ionized calcium binding adaptor molecule 1 (Iba-1) (Fig. 3a). Image analysis of the GFAP and Iba-1 staining showed a statistically significant increase in astrocytosis and microgliosis in the hippocampus of Tg2576 mice subjected to 2 blood transfusions (Fig. 3b, c). Analysis of the relationship between amyloid deposition (measured by ThS staining) and astrogliosis in different groups of animals showed a positive correlation (Fig. 3d), suggesting that astroglial reaction was elicited in response to Aβ accumulation, as is commonly the case in humans and transgenic mouse models of AD.

### Induction of amyloid and associated damage is not mediated by an inflammatory reaction to blood infusions

**Fig. 4:**
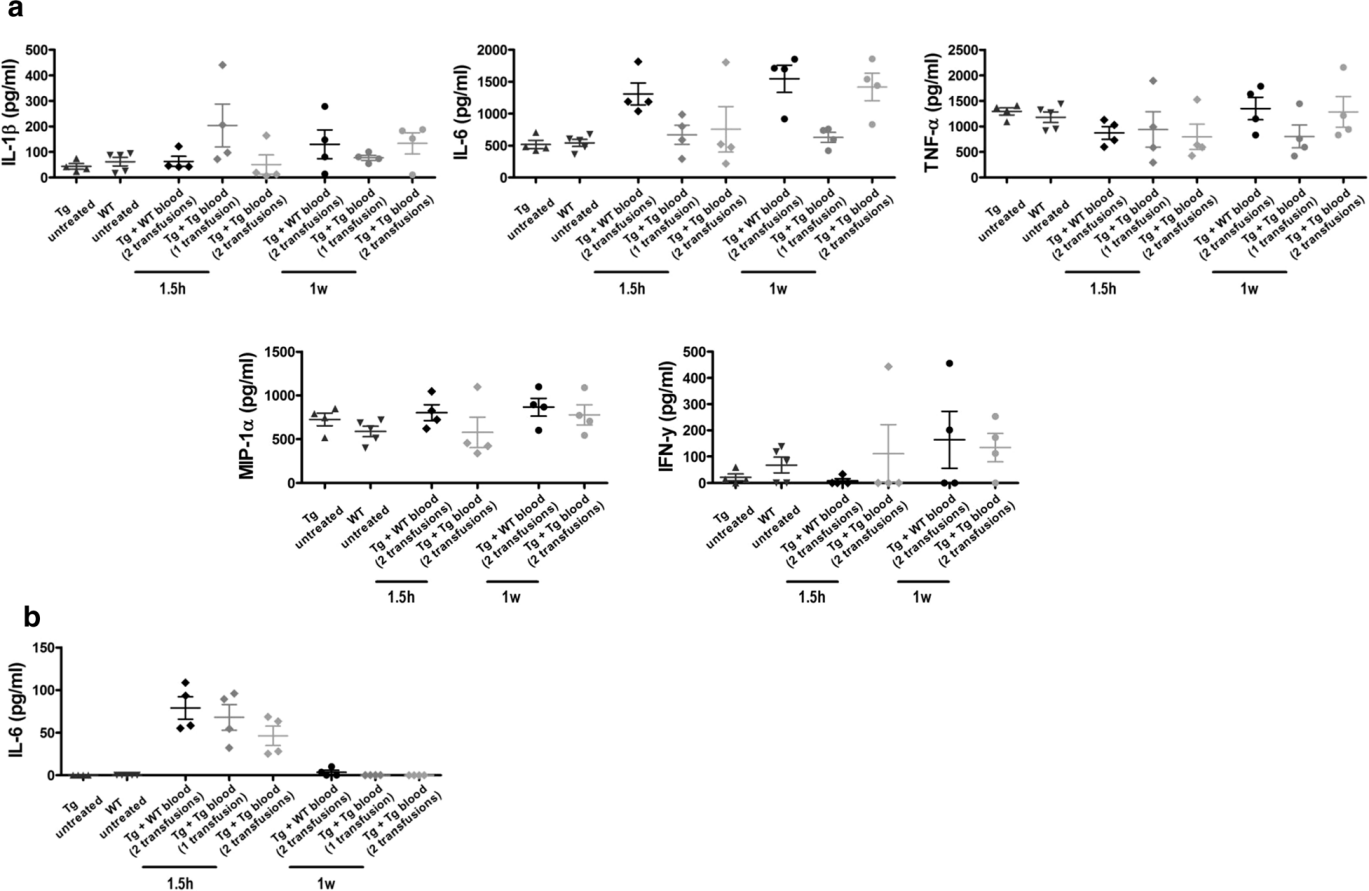
Amyloid induction by blood infusion is not mediated by acute systemic inflammation. The development of a systemic inflammatory response to blood infusions was measured in peripheral blood mononuclear cells (PBMC) (**a**) and plasma (**b**) from animals subjected to the different treatments (n = 4). Various pro-inflammatory cytokines (IL-1β, IL-6, TNFα, MIP-1α, IFN-γ, IL-10 and MCP-1) were measured 1.5 h or 7 days after blood administration to evaluate acute and chronic inflammatory changes. Release of these cytokines from PBMC was measured after stimulation with lipopolysaccharide, as described in “Materials and methods” section. In PBMC, IL-10 and MCP-1 were not detectable above the background and in plasma, only IL-6 was detectable above the background levels. Statistical analyses showed that blood administration produced a significant activation of these cytokines; however no differences were observed between WT and transgenic blood

To evaluate whether the increase of amyloid deposition observed after 2 blood transfusions was mediated by a systemic inflammatory response, we performed a detailed measurement of various inflammatory markers (IL-1β, IL-6, TNFα, MIP-1α, IFN-γ, IL-10 and MCP-1) in plasma and peripheral blood mononuclear cells (PBMC) from animals subjected to the different treatments. Cytokines were measured 1.5 h or 7 days after blood administration to evaluate acute and chronic inflammatory changes. Release of these inflammatory markers from PBMC was measured after stimulation with lipopolysaccharide (Fig. 4a). The results did not show conclusive differences on the levels of any of these proteins released by PBMC at the times measured between Tg2576 mice treated with WT- or Tg-blood. We also measured the levels of these cytokines in plasma. Only IL-6 was detectable beyond the background levels at 1.5 h after blood administration, suggesting that the treatment does indeed induce an acute inflammatory response (Fig. 4b). However, the levels of this cytokine were not different in transgenic mice exposed to WT blood or to 1 or 2 transfusions with transgenic blood. No detectable levels of this cytokine were observed 1 week after treatment. Also, the IL-6 levels in plasma after 1 or 2 blood transfusions were not different, suggesting the lack of an accumulative effect. Altogether, these results indicate that although blood transfusion induced a mild and acute inflammatory response, there were no differences depending on whether the blood came from wild type or transgenic mice. These findings suggest that the acceleration of brain amyloidosis by blood infusions was not dependent on a systemic inflammatory response. However, since we only measured a limited number of the possible inflammatory markers, we cannot completely rule out that elevation on other proteins associated with inflammation may be responsible for the effects observed.

### Acceleration of brain Aβ deposition was reproducible in different experimental models

To study the reproducibility of the induction of AD-like pathological alterations by blood infusions, we performed several independent experiments using different animal models and distinct inocula, including blood from transgenic mice of different ages, fractionated blood, intra-venous inoculation with brain extracts and recombinant Aβ aggregates. As shown in Table 1 several of the conditions tested accelerated pathology in a large proportion of the animals studied. For example, Experiment 4 in Table 1 and Additional file 1: Figure S2 summarizes the results of a study done using a different transgenic mouse model carrying the human mutant versions of APP (with the Swedish mutation) and presenilin-1 (PSEN1, with the delta-E9 mutation). These animals develop Aβ deposits starting at around 4–5 months of age [20]. Whole blood from 12 months old APP/PS1 or age-matched WT littermates was infused intra-venously every week for 3 consecutive weeks into groups of young (60 days old) APP/PS1 mice. Animals were sacrificed at 150 days old and brain collected for histopathological analyses. The results showed that 80% of treated mice had a significantly higher load of amyloid deposits than the controls transfused with WT-blood (Table 1 and Additional file 1: Figure S2). This data indicates that the effect of blood transfusion is not restricted to Tg2576 animals used for the previous studies. Experiment 8 in Table 1 and Additional file 1: Figure S6 describes the results obtained when 10% brain extracts from 12 months old Tg2576 exhibiting substantial amyloid pathology were injected intra-venously into young Tg2576 mice. Unfortunately, most of the animals used for this experiment (18 out of 20) died soon after injection, most likely because of stroke events produced by small pieces of debris present in the brain extract. The two surviving animals showed a substantially higher burden of amyloid deposition in the brain suggesting that Aβ aggregates administered in blood can reach the brain and seed amyloid pathology. An interesting set of results were obtained in studies in which animals received blood from donors of different ages which had distinct stages of amyloid pathology in the brain. When 50 days old Tg2576 animals received blood infusions from young Tg2576 mice (2–4 months old) which did not exhibit any accumulation of cerebral Aβ aggregates, only 1 out of 8 recipient mice showed increased amyloid pathology (Experiment 5 in Table 1 and Additional file 1: Figure S3). In a similar experiment in which animals received blood from Tg2576 mice collected at the time when these animals begin to show spontaneous amyloid pathology (7–9 months old), 3/7 mice developed accelerated amyloid deposition albeit differences were not significant between the groups (Experiment 6 in Table 1 and Additional file 1: Figure S4). As shown in Fig. 1, a large proportion (80–100% depending on the parameter measured) of Tg2576 mice transfused with blood from 12 to 14 months old donors with established amyloid pathology, developed significantly higher pathological changes compared with controls (Experiment 1 in Table 1). Surprisingly, when animals were transfused with blood coming from old Tg2576 animals (15–18 months of age), exhibiting massive accumulation of amyloid deposits, only 1/6 mice developed exacerbated pathology compared to controls (Experiment 7 in Table 1 and Additional file 1: Figure S5). A possible interpretation for this puzzling result is that at severe stages of amyloid deposition, most of smaller and freely circulating, soluble Aβ species are encapsulated in plaques. This interpretation finds support in the well-established observation that total levels of Aβ_42_ are lower in cerebrospinal fluid (CSF) of AD patients than in controls [26]. Indeed, *post*-*mortem* studies found a good negative correlation between the extent of cerebral amyloid pathology and the decrease on CSF circulating Aβ_42_ [27]. In the same line, it has also been reported that significantly high and variable amounts of oligomeric/aggregated Aβ in blood of human AD patients (compared to controls) decreased over time during the progression of the disease [28].

### Amyloid-inducing activity is present in the plasma fraction

**Fig. 5:**
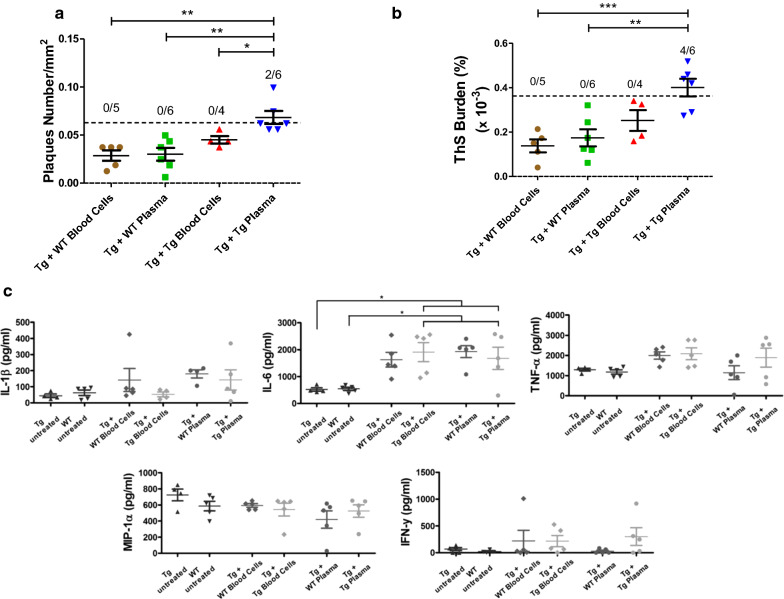
Transfusion with isolated blood plasma increases Aβ deposition in Tg2576 mice. Groups of young Tg2576 animals were intravenously challenged with 300 µL of blood cells or plasma obtained from old Tg2576 mice or WT littermates. The number of amyloid plaques in experimental and control animals was counted and expressed as number of plaques per mm^2^ of brain area analyzed (**a**) and ThS burden was measured as the ThS stained area in relation to the total brain area (cortex and hippocampus) analyzed (**b**) and expressed as percentage. *P < 0.05, **P < 0.01, ***P < 0.001 based on ANOVA followed by the Tukey’s multiple comparison post hoc test. The dotted line in panels **a** and **b** represents the threshold value used to calculate attack rate, i.e. the proportion of individual animals having significantly higher pathology than controls as explained in “Materials and methods” section. The numbers on top indicate the ratio of animals over the threshold/total number of animals in the group. **C** Levels of inflammatory markers at the experimental endpoint in animals subjected to transfusion with blood fractions. Various inflammatory markers (IL-1β, IL-6, TNFα, MIP-1α, IFN-γ, IL-10 and MCP-1) were measured in PBMC and plasma. Unfortunately none of the markers were observed above the background levels in plasma and only IL-1β, IL-6, TNFα, MIP-1α and IFN-γ were detectable in LPS stimulated PBMC. Statistical analyses showed that blood administration produced a significant increase of the levels of only IL-6. However, no differences were observed between administration of WT or transgenic blood

Next, to investigate the fraction of the blood that carries the activity responsible for accelerating the pathology in the brain, we performed an experiment in which Tg2576 animals were transfused in the same manner and frequency as the study shown in Fig. 1, but using isolated fractions of blood cells and plasma. The result shows that only transfusion with plasma produced a statistically significant increase in Aβ deposits (Fig. 5a, b). Additionally, in order to further evaluate a putative systemic inflammatory response after infusion of blood fractions, we measured the levels of various inflammatory markers in PBMC collected at the time animals were sacrificed for histological and biochemical studies. Administration of blood fractions did not produce a significant elevation of the levels of most of the inflammatory markers tested (IL-1β, TNFα, MIP-1α and IFN-γ), but only increased levels of IL-6 were detected in animals subjected to blood transfusion (Fig. 5c). However, as before, the results did not show any differences between animals treated with either WT or transgenic blood fractions.

### Intra-venous administration of purified Aβ fibrils increase amyloid deposition in the brain of APP/PS1 mice

**Fig. 6:**
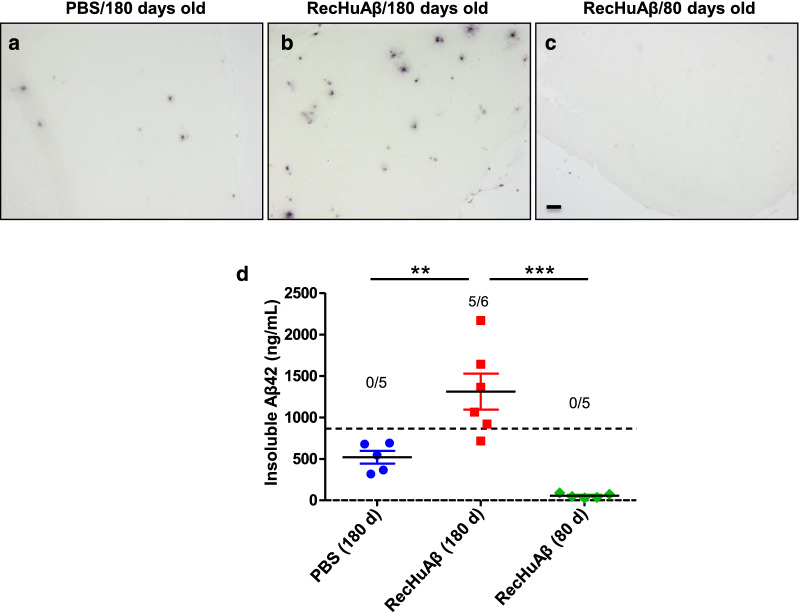
Intra-venous administration of pure recombinant Aβ aggregates accelerates cerebral amyloid pathology. Young APP/PS1 double transgenic mice (30 days old) were injected i.v. with a preparation containing in vitro generated Aβ aggregates (200 µL), as described in the “Materials and Methods” section. Animals were injected once a week for 3 consecutive weeks and sacrificed at 180 days old. As controls, mice were injected i.v. with the same volume of PBS. To study the fate of the inoculum at short time periods, a group of animals injected with Aβ aggregates were sacrificed 50 days after the last injection. **a**–**c** Representative pictures of 4G8 stained brain slices from experimental [RecHuAβ/80 days old (**a**)] or control groups [PBS/180 days old (**b**) and RecHuAβ/80 days old] mice. Scale bar at the left-bottom of the “RecHuAβ/80 days old” picture represents 100 µm and applies to all pictures. **d** The concentration of PBS insoluble Aβ_42_ in the brain of these animals was measured by ELISA. Symbols show the values of individual animals and the dotted line represents the threshold value used to calculate attack rate (in regards to PBS injected mice). The numbers on top indicate the ratio of animals over the threshold/total number of animals in the group. Data was analyzed by one-way ANOVA followed by the Tukey’s multiple comparison post hoc test. *P < 0.05; ***P < 0.001

To further support the idea that the acceleration of cerebral amyloid pathology by blood transfusion was mediated by a seeding activity of Aβ aggregates, we attempted to immune-deplete plasma samples using a cocktail of antibodies specific for the sequence or conformation of Aβ oligomers. Unfortunately the results of this experiment were inconclusive likely because it is unknown the nature of Aβ oligomers in blood and which antibodies to use for depletion. Further complications are the small effect observed with plasma which is substantially lower than total blood (compare Figs. 1 and 5) and the relatively large removal of Aβ with control/non-conjugated beads alone [29]. Another way to further support the conclusion that the active principle in inducing cerebral amyloid deposition upon transfusion with Tg-blood was the Aβ aggregates themselves is to analyze the effect of intra-venous injection of in vitro generated Aβ aggregates prepared from pure recombinant proteins. It has been shown that synthetic Aβ aggregates can induce amyloid deposition when administered directly into the brain [14]. For our experiments, young transgenic mice were injected into the blood with a preparation containing in vitro produced Aβ aggregates. For this purpose pure recombinant Aβ_42_ was incubated in PBS for 5 days to generate a heterogeneous mixture of various Aβ aggregates, including small and large oligomers, protofibrils and fibrils. This material can efficiently seed Aβ aggregation in vitro even at high dilutions [21]. Aliquots of this preparation were injected i.v. 3 times into APP/PS1 mice (weekly, starting at 30 days old). Animals were sacrificed at 180 days of age and brains analyzed for the presence of amyloid deposits. Histological analysis using an anti-Aβ antibody showed a significantly higher amount of amyloid plaques in mice inoculated with synthetic Aβ aggregates compared to PBS treated mice (Fig. 6a–c). Indeed, 5 out of 6 mice inoculated i.v. with recombinant aggregates developed a substantially more severe amyloid pathology than controls (Fig. 6d). To examine whether the higher load of cerebral amyloid observed in these animals reflected simply the deposition of the aggregates injected, we sacrificed a group of animals 30 days after the last injection (~ 80 days of age). No detectable presence of amyloid deposits was observed in these mice (Fig. 6a, right panel), indicating that the larger presence of amyloid plaques in the group analyzed at 180 days old was due to seeding and aggregation of endogenous Aβ. A similar conclusion was obtained in a report in which aggregates of a chemically modified (isoAsp7) version of Aβ_1-42_ were repetitively injected intra-venously into transgenic mice [30].

## Discussion

Several studies have demonstrated that Aβ deposition can be induced in vivo in diverse animal models by injection of AD brain homogenates carrying Aβ aggregates in a similar manner as prions transmit prion diseases [11–13, 31]. The initial studies were done by injecting the material intra-cerebrally; however, reports from the Jucker’s group showed that intra-peritoneal (i.p.) administration of brain extracts containing Aβ aggregates was also able to induce cerebral pathology, albeit with a lower efficiency [13]. Interestingly, the extent of induction by i.p administration was dependent on the level of Aβ expression in the brain but not in the periphery [32]. The distribution of Aβ deposits in brain was consistent with the entry of Aβ seeds at multiple brain locations [32]. Intra-peritoneally injected Aβ was detectable in blood, particularly associated to monocytes and some peripheral tissues (liver, spleen) for up to 30 days after injection [32]. These results as well as our current findings suggest that peripherally administered Aβ aggregates can reach the brain and seed cerebral amyloidosis. This is not entirely surprising considering previous reports showing that Aβ can be efficiently transported across the blood brain barrier [33–36] and peripheral administration of labeled Aβ can be retrieved bound to amyloid plaques in the brain [35, 37]. Previous studies from us and others have also shown that the active principle responsible for inducing amyloid pathology are the Aβ aggregates themselves [12, 14, 29] and that the process can be initiated with a small quantity of preformed aggregates [38, 39]. Indeed, intra-cerebral injection of as little as a million fold dilution of brain containing Aβ aggregates is sufficient to significantly accelerate amyloid deposition [38].

The main contribution of our current study is to show that blood from animals with established cerebral amyloid pathology can accelerate Aβ deposition and associated neuropathological alterations when i.v. infused into young recipients. Aβ levels in the blood of Tg2576 mice have been extensively reported [40–42]. Both Aβ_40_ and Aβ_42_ peptides are present in the blood of these mice at the nM range, and their concentration decrease as brain pathology progresses. Although not as high as the acceleration of brain amyloidosis followed by intra-cerebral or intra-peritoneal administrations of Aβ aggregates, our results show that blood from different sources (ages and mouse models) can accelerate AD-like phenotypes. As shown in Fig. 1, three independent methods using different principles (immunohistochemistry, fluorescence staining with amyloid-binding dye and biochemical studies by ELISA) used to assess brain amyloidosis in control and experimental groups provided similar conclusions, supporting the robustness of the results obtained. However, despite the many control groups used in our studies and our data showing that administration of purified/recombinant Aβ aggregates into blood produces a similar result, we cannot completely rule out that another component in Tg-blood might be responsible for the induction of pathological alterations. This alternative interpretation find some support in our data showing a trend, although not significant, for increased brain amyloidosis on animals receiving WT blood or 1 Tg blood transfusion compared to untreated mice. Further support for this alternative explanation comes from the studies by Villeda and co-workers [43] who suggest that blood-borne factors present in the systemic milieu of old mice negatively regulate neurogenesis and cognitive function, accelerating aging-associated phenotypes. Considering that aging is the main risk factor for brain Aβ deposition, it is reasonable to speculate that deleterious components, other than Aβ itself, present in the blood of old Tg2576 mice may be responsible for the effects observed in this study. Nevertheless, our current results argue against this possibility since blood from the oldest Tg2576 donors resulted in similar or even lower attack rates in terms of pathological changes when compared to mice receiving blood from younger blood donors. From the host’s side, an additional possibility to explain our results is that untested inflammatory components reactive to specific molecules present on Tg blood may be responsible for the effects observed. Nonetheless, this is unlikely since recipient animals were generated in the same genetic background as blood donors. The use of appropriate controls (blood from age matched WT littermates), the intravenous administration of purified/recombinant Aβ aggregates, and the known fact that Aβ species (including oligomers) can cross the blood–brain barrier strongly suggest that Aβ aggregates present in the blood of donor Tg2576 mice are responsible for the effects described in this article. Nevertheless, a detailed assessment of all these possibilities, including the role of factors other than Aβ aggregates (such as CCL2 and CCL11 [43]) in the acceleration of brain amyloidosis needs to be further investigated.

As mentioned, our findings indicate that some pathological abnormalities typical of AD can be accelerated in animal models by blood infusions, a frequent medical practice that has been shown to transmit prion disease in animals and humans [17, 18]. However, it is important to highlight that our experiments were done in animal models that were created by highly over-expressing a mutant human protein and they only reproduce in part some of the aspects of AD. Therefore, the results obtained could be largely dependent on the overexpression of the gene in brain and peripheral tissues. Thus, extrapolation of our results to humans should not be done without further experiments. At this time, there is no epidemiological evidence for the transmissibility of AD in humans by blood transfusions. Indeed, two small case-control studies reported that a history of blood transfusion is not associated with increased risk of AD [44, 45]. Nevertheless, recent studies showing the presence of Aβ pathology on brain samples from iatrogenic cases of Creutzfeldt-Jakob disease [46–48] have provided indirect support for human-to-human transmission of Aβ misfolding. These studies showed that brain Aβ deposition was present at young ages (when it was not expected to appear) and deposition was strongly associated with blood vessels, similarly to what is observed for Aβ induced by peripheral routes [13]. In light of these recent studies, it is essential to fully understand the mechanisms implicated in seeding of Aβ deposition, the effective routes of transmission and the putative sources of Aβ seeds.

Surprisingly, blood from Tg2576 donors obtained at severe stages of cerebral Aβ pathology did not cause the larger effect expected in terms of acceleration of AD-like lesions. A possible interpretation for this puzzling result is that at severe stages of amyloid deposition most of smaller and freely circulating/soluble Aβ species are encapsulated in plaques. This interpretation finds support in the well-established observation that total levels of Aβ_42_ are lower in cerebrospinal fluid (CSF) of AD patients than in controls [26]. As mentioned above, total Aβ levels in blood plasma of Tg2576 mice decrease as brain amyloidosis increase [40]. Indeed, *post*-*mortem* studies found a good negative correlation between the extent of cerebral amyloid pathology and the decrease on CSF circulating Aβ_42_ [27]. In the same line, it has also been reported that significantly high and variable amounts of oligomeric/aggregated Aβ in blood of human AD patients (compared to controls) decreased over time during the progression of the disease [28]. An alternative interpretation of these findings is that other molecules able to accelerate pathological changes in the brain (besides Aβ) and differentially expressed in the blood of Tg2576 mice at different ages peaked at 12-14 months old (or conversely, neuroprotective factors importantly decrease at this specific age in these mice). The presence and levels of Aβ aggregates and other molecules associated with pathological changes in the blood of Tg2576 mice at different ages is currently under investigation.

An unaddressed topic in our research deals with the role of sex in brain amyloid modulation by blood infusions. It has long been established the association between female sex and increased AD incidence [49]. This has also been observed in animal models of brain amyloidosis [50]. The overall sex distribution in this experiment was balanced although some groups were clearly enriched in a single sex (Additional file 1: Table S1). Nevertheless, relevant groups such as “Tg + WT blood (2 transfusions)” and “Tg + Tg blood (2 transfusions)” (Fig. 1) had identical sex distributions and validated our conclusions. It is important to consider that sex differences in Tg2576 mice manifest at advanced ages (17 months old [50]), way later than our experimental endpoints (~ 8–10 months old). Analysis of individual data distribution by sex suggest that this factor did not played a major role in the results presented in this study. However, future research will address whether sex affects blood-induced amyloidosis (from the donor and recipient perspectives).

Interestingly, no CAA was observed for any of the blood infusion experiments described in this article. However, CAA was observed in APP/PS1 mice injected with recombinant Aβ fibrils, in agreement with previous reports [16]. To explain these results, we hypothesize that small Aβ oligomers circulating in blood of Tg2576 or APP/PS1 mice are able to cross the blood brain barrier of recipient animals. Later on, these oligomers could diffuse across the brain parenchyma and seed amyloid pathology. On the contrary, larger Aβ fibrils will have increased difficulties to cross the blood brain barrier. In that sense, they will remain in blood vessels and seed in situ, promoting CAA. Regardless of this hypothetical scenarios, it is uncertain whether APP/PS1 leads to increased CAA after seeding compared to Tg2576. Importantly, vascular amyloid deposition have been observed in several cases of possible iatrogenic Aβ misfolding transmission [46, 48, 51, 52].

## Conclusions

The main conclusion of this article is that blood (especially plasma) from Tg2576 mice carries biologically active seeds or other components (such as specific cytokines, etc.) that accelerate brain amyloidosis. This may open new avenues of research, exploring the role of blood in the progression of brain Aβ amyloidogenesis. Consequently, the identification of such components circulating in blood could represent an attractive target for AD diagnosis. In the same line, our data reinforce the idea that removal of these components from peripheral circulation may delay brain pathology [53], in an easier and less invasive way compared to interventions at the brain level.

## Supplementary information

**Additional file 1: Figure S1.** Experimental strategy for animal treatments by intravenous infusions. Schematic summary of treatments in animals. **A)** applies to Experiments 1 and 2; **B)** applies to experiment 3; **C)** applies to Experiment 4; **D)** applies to Experiment 5, 6 and 7; and **E)** applies to experiment 8. **Figure S2.** APP/PS1 blood infusion increases cerebral Aβ deposition in younger animals carrying the same transgene (Experiment 4). A similar experiment to the one shown in Figure 1 was independently performed using APP/PS1 mice as explained in Materials and Methods. Image analysis was used to quantify the burden of 4G8-stained plaques in the cerebral cortex of mice receiving blood from 12 months old APP/PS1 (red squares) or age-matched WT littermates (black circles). The values were expressed as the mean ± SEM (n=4-5). Data was analyzed by Student’s t-test. * P<0.05. **Figure S3.** Blood transfusion experiment using blood from 2-4 months old Tg2576 donors (Experiment 5). Young Tg2576 mice received blood from Tg2576 (red squares) or WT (black circles) mice ranging from 2 to 4.3 months old as explained in Materials and Methods. PBS-insoluble levels of Aβ_42_ measured by ELISA were assessed in the brain of blood-treated mice at the endpoint. The horizontal/dotted line was used to define the attack rate as explained in the body of the manuscript. Data was analyzed by Student’s t-test. n.s; no-significant. **Figure S4.** Blood transfusion experiment using blood from 7-9 months old Tg2576 donors (Experiment 6). Young Tg2576 mice received blood from Tg2576 (red squares) or WT (black circles) mice ranging from 7.9 to 9.2 months old as explained in Materials and Methods. PBS-insoluble levels of Aβ_42_ measured by ELISA were assessed in the brain of blood-treated mice at the endpoint. The horizontal/dotted line was used to define the attack rate as explained in the body of the manuscript. Data was analyzed by Student’s t-test. n.s; no-significant. **Figure S5.** Blood transfusion experiment using blood from 15-20 months old Tg2576 donors (Experiment 7). Young Tg2576 mice received blood from Tg2576 (red squares) or WT (black circles) mice ranging from 15.5 to 20 months old as explained in Materials and Methods. PBS-insoluble levels of Aβ_42_ measured by ELISA were assessed in the brain of experimental mice at the endpoint. The horizontal/dotted line was used to define the attack rate as explained in the body of the manuscript. Data was analyzed by Student’s t-test. n.s; no-significant. **Figure S6.** Intravenous administration of Tg2576 brain homogenate in young Tg2576 mice (Experiment 8). Young Tg2576 mice were intravenously injected with a 10% w/v Tg2576 brain homogenate (red squares) obtained from a 365 days old Tg2576 mice. Image analysis was used to quantify the burden of 4G8-stained plaques in the cerebral cortex and hippocampus. Mice receiving blood from age matched WT mice (black circles) were used as controls to define attack rates (same as depicted in Figure 1) as defined in the body of the manuscript. **Table S1**. Sex distribution of mice. The table below list the number of female and male mice used for each experiment included in this manuscript.

## Data Availability

The datasets during and/or analyzed during the current study available from the corresponding authors on reasonable request.
