# Supplementary material for: Infusion of blood from mice displaying cerebral amyloidosis accelerates amyloid pathology in animal models of Alzheimer’s disease
Source: Acta Neuropathol Commun. 2020 Dec 7;8:213. doi: 10.1186/s40478-020-01087-1 (PMC7720397; doi:10.1186/s40478-020-01087-1)

Supplemental Table 1: Sex distribution of mice

Experiment	Group	Male	Female
**Experiment 1**	Tg untreated	2	11
	Tg+ WT blood (2 transfusions)	1	4
	Tg + Tg blood (1 transfusion)	6	0
	Tg + Tg blood (2 transfusion)	1	4
	WT + Tg blood (2 transfusions)	6	0
**Experiment 2**	Tg2576 + WT blood cells	0	5
	Tg2576 + WT Plasma	4	2
	Tg2576 + Tg blood cells	2	2
	Tg2576 + Tg Plasma	3	2
**Experiment 3**	PBS	4	1
	RecHuAb (6 m)	2	4
	RecHuAb (2.5 m)	3	2
**Experiment 4**	APP/PSEN (WT blood)	1	3
	APP/PSEN (Tg blood)	1	4
**Experiment 5**	Tg+ WT blood (2 transfusions)	2	2
	Tg + Tg blood (2 transfusion)	2	6
**Experiment 6**	Tg+ WT blood (2 transfusions)	3	2
	Tg + Tg blood (2 transfusion)	3	4
**Experiment 7**	Tg+ WT blood (2 transfusions)	1	4
	Tg + Tg blood (2 transfusion)	4	2
**Experiment 8**	Tg+ WT blood (2 transfusions)[a]	1	4
	Tg + 10% Tg brain homogenate	1	1

[a] This group is the same as the one presented in Experiment 1

Supplemental Figure 1

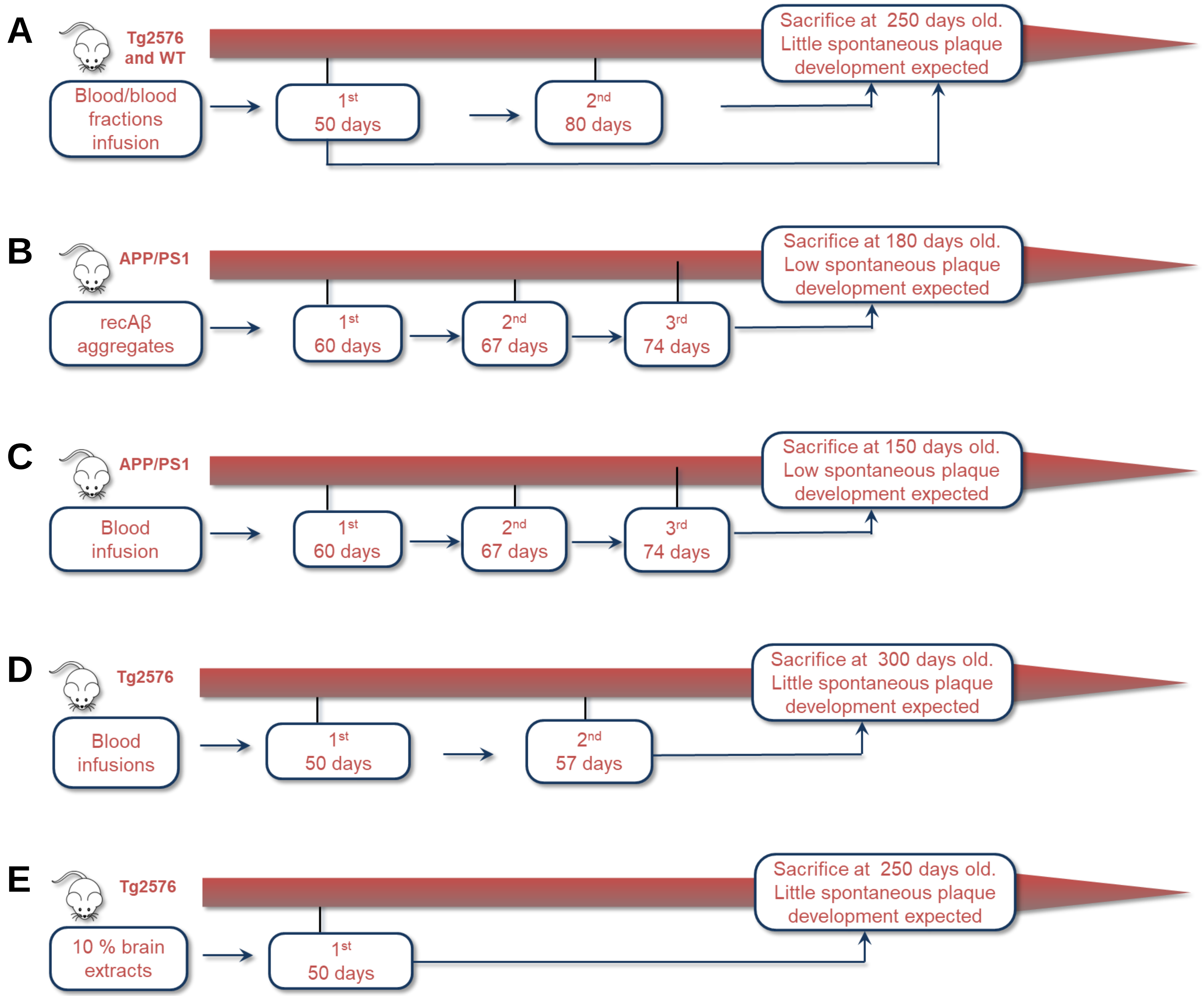

Supplemental Figure 2

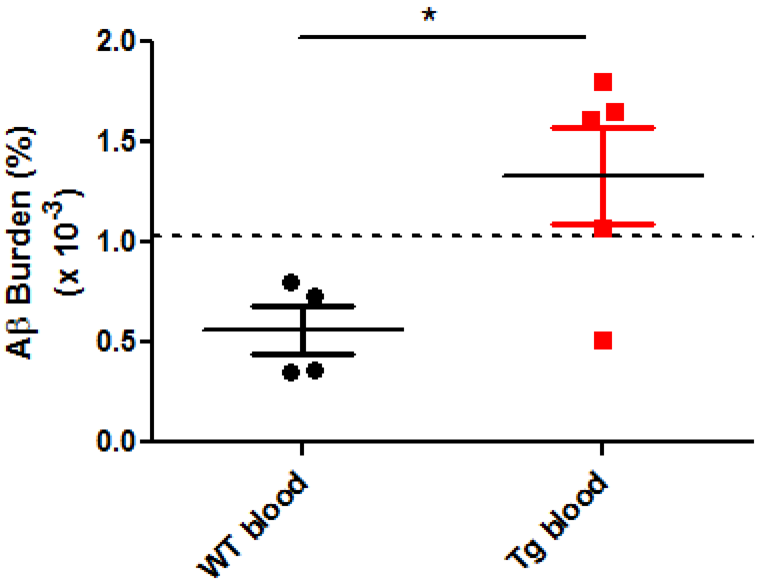

Supplemental Figure 3

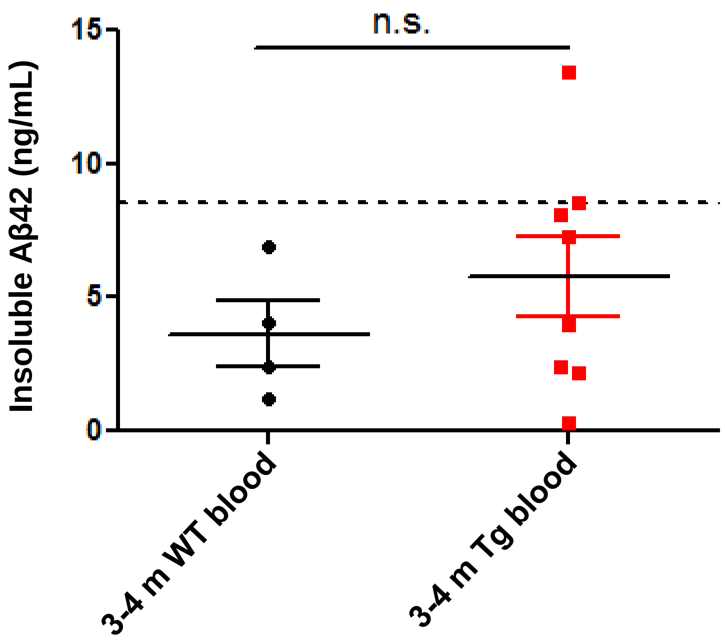

Supplemental Figure 4

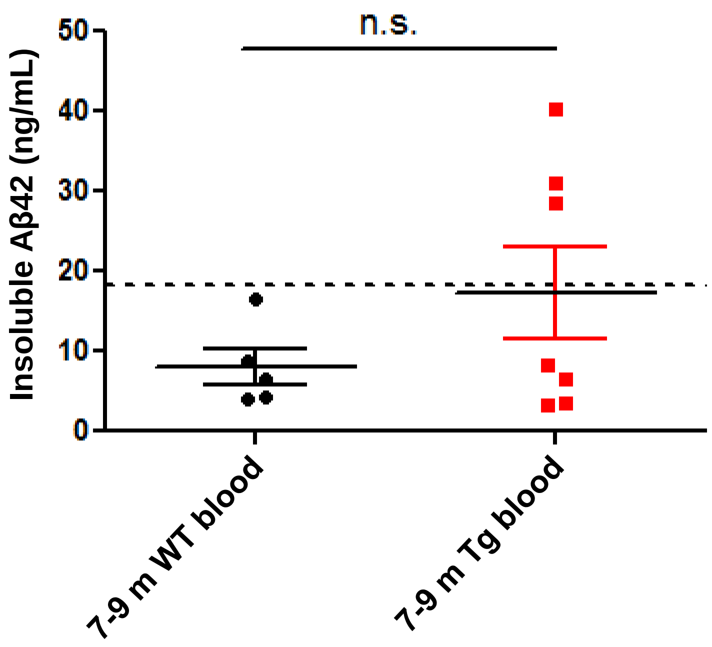

Supplemental Figure 5

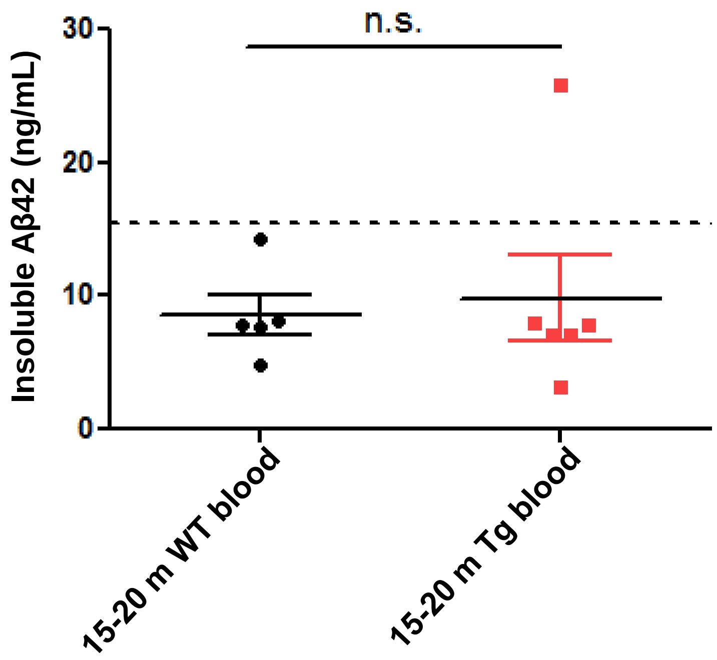

Supplemental Figure 6

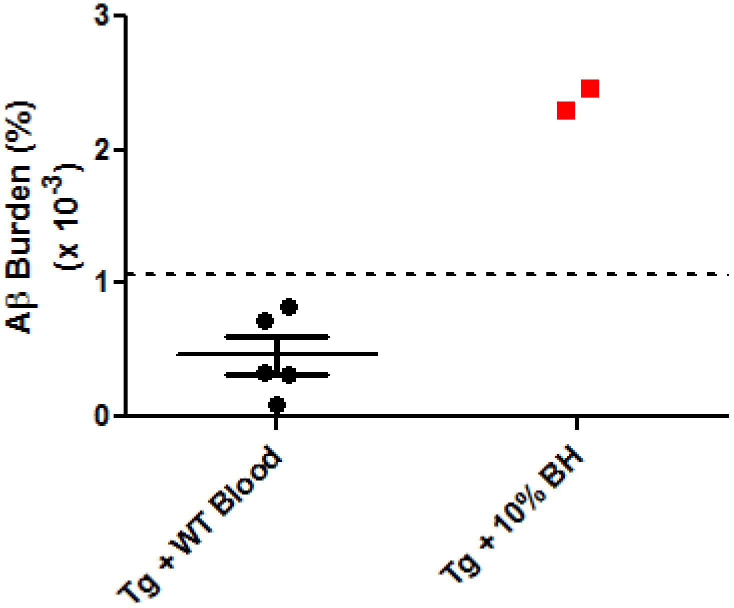

Supplement: Supplementary file 1 — Additional file 1: Figure S1. Experimental strategy for animal treatments by intravenous infusions. Schematic summary of treatments in animals. A) applies to Experiments 1 and 2; B) applies to experiment 3; C) applies to Experiment 4; D) applies to Experiment 5, 6 and 7; and E) applies to experiment 8. Figure S2. APP/PS1 blood infusion increases cerebral Aβ deposition in younger animals carrying the same transgene (Experiment 4). A similar experiment to the one shown in Figure 1 was independently performed using APP/PS1 mice as explained in Materials and Methods. Image analysis was used to quantify the burden of 4G8-stained plaques in the cerebral cortex of mice receiving blood from 12 months old APP/PS1 (red squares) or age-matched WT littermates (black circles). The values were expressed as the mean ± SEM (n=4-5). Data was analyzed by Student’s t-test. * P<0.05. Figure S3. Blood transfusion experiment using blood from 2-4 months old Tg2576 donors (Experiment 5). Young Tg2576 mice received blood from Tg2576 (red squares) or WT (black circles) mice ranging from 2 to 4.3 months old as explained in Materials and Methods. PBS-insoluble levels of Aβ42 measured by ELISA were assessed in the brain of blood-treated mice at the endpoint. The horizontal/dotted line was used to define the attack rate as explained in the body of the manuscript. Data was analyzed by Student’s t-test. n.s; no-significant. Figure S4. Blood transfusion experiment using blood from 7-9 months old Tg2576 donors (Experiment 6). Young Tg2576 mice received blood from Tg2576 (red squares) or WT (black circles) mice ranging from 7.9 to 9.2 months old as explained in Materials and Methods. PBS-insoluble levels of Aβ42 measured by ELISA were assessed in the brain of blood-treated mice at the endpoint. The horizontal/dotted line was used to define the attack rate as explained in the body of the manuscript. Data was analyzed by Student’s t-test. n.s; no-significant. Figure S5. Blood transfusion experiment usi [file 40478_2020_1087_MOESM1_ESM.pdf]
